# Heterogeneity analysis of Metastasis Associated in Colon Cancer 1 (MACC1) for survival prognosis of colorectal cancer patients: a retrospective cohort study

**DOI:** 10.1186/s12885-015-1150-z

**Published:** 2015-03-21

**Authors:** Viktor H Koelzer, Pia Herrmann, Inti Zlobec, Eva Karamitopoulou, Alessandro Lugli, Ulrike Stein

**Affiliations:** 1 Translational Research Unit (TRU), Institute of Pathology, University of Bern, Murtenstrasse 31, Bern, CH-3010, Switzerland; 2 Clinical Pathology Division, Institute of Pathology, University of Bern, Murtenstrasse 31, Bern, CH-3010, Switzerland; 3 Department of Translational Oncology of Solid Tumors, Experimental and Clinical Research Center, Charité University Medicine Berlin and Max-Delbrück-Center for Molecular Medicine, Robert-Rössle-Strasse 10, D-13125, Berlin, Germany; 4 German Cancer Consortium (DKTK), Im Neuenheimer Feld 280, D-69120, Heidelberg, Germany

**Keywords:** MACC1, Biomarker, Tumor budding, Colorectal cancer, Prognostic factor, Metastasis

## Abstract

**Background:**

Metastasis of colorectal cancer (CRC) is directly linked to patient survival. We previously identified the novel gene Metastasis Associated in Colon Cancer 1 (MACC1) in CRC and demonstrated its importance as metastasis inducer and prognostic biomarker. Here, we investigate the geographic expression pattern of MACC1 in colorectal adenocarcinoma and tumor buds in correlation with clinicopathological and molecular features for improvement of survival prognosis.

**Methods:**

We performed geographic MACC1 expression analysis in tumor center, invasive front and tumor buds on whole tissue sections of 187 well-characterized CRCs by immunohistochemistry. MACC1 expression in each geographic zone was analyzed with Mismatch repair (MMR)-status, BRAF/KRAS-mutations and CpG-island methylation.

**Results:**

MACC1 was significantly overexpressed in tumor tissue as compared to normal mucosa (p < 0.001). Within colorectal adenocarcinomas, a significant increase of MACC1 from tumor center to front (p = 0.0012) was detected. MACC1 was highly overexpressed in 55% tumor budding cells. Independent of geographic location, MACC1 predicted advanced pT and pN-stages, high grade tumor budding, venous and lymphatic invasion (p < 0.05). High MACC1 expression at the invasive front was decisive for prediction of metastasis (p = 0.0223) and poor survival (p = 0.0217). The geographic pattern of MACC1 did not correlate with MMR-status, BRAF/KRAS-mutations or CpG-island methylation.

**Conclusion:**

MACC1 is differentially expressed in CRC. At the invasive front, MACC1 expression predicts best aggressive clinicopathological features, tumor budding, metastasis formation and poor survival outcome.

**Electronic supplementary material:**

The online version of this article (doi:10.1186/s12885-015-1150-z) contains supplementary material, which is available to authorized users.

## Background

Colorectal cancer (CRC) is still one of the most frequent malignancies in the Western world with more than 1 million new cases every year. The life time risk to suffer from CRC is about 5% in developed countries [1,2]. Metastasis of primary colorectal tumors is directly linked to patient survival and accounts for about 90% of patient deaths. About half of the subjects with CRC can be cured by surgery and multimodal treatment, but therapy options are limited particularly for metastasized patients. This is demonstrated by 5-year-survival rates of higher than 90% for early stage patients, 65% for patients with regional lymph node metastases, and less than 10% in patients with metastatic disease [2]. Synchronous distant metastases were already observed in about 30% of CRC patients, and at least a further third will develop metachronous metastases later, despite primary treatment with curative intention [2]. Therefore, development of distant metastases is the most crucial and lethal event during the disease course, critically limiting therapy options. Since current clinical and histopathological classifications and molecular markers are not sufficient for prediction of metastasis, the development of biomarkers for the early and precise identification of patients at high-risk for metastasis at early stages of the disease is of utmost importance.

We identified the novel gene Metastasis Associated in Colon Cancer 1, MACC1, based on human colon cancer specimens [3]. In cell culture, MACC1 drives proliferation, migration, invasion, wound healing and dissemination and regulates genes transcriptionally important for metastasis, e.g. the receptor tyrosine kinase MET. It is crucially involved in fundamental biological processes, e.g. apoptosis and epithelial-mesenchymal transition (EMT), via pathways such as the HGF/MET/MACC1 axis. In several xenograft mouse models, MACC1 induces tumor progression and metastasis [3,4].

In CRC patients, MACC1 is a tumor stage-independent predictor for metastasis and survival, and allows early identification of high-risk cases [4-6]. Importantly, MACC1 has also been identified as a valuable biomarker in carcinomas of the gastrointestinal tract such as gastric [7], esophagus [8], pancreatic [9] and hepatobiliary [10-12] as well as in carcinomas of the lung [13-15], ovaries [16], breast [17,18], upper urothelial tract [19], nasopharynx [20], malignant glioma [21,22] and osteosarcomas [23]. Remarkably, MACC1 levels consistently correlated with tumor progression, development of metastasis and patient survival in this broad range of solid tumor types, making MACC1 a decisive driver for disease progression (reviewed in [24]). The predictive value of MACC1 for therapy response was demonstrated in rectal, pancreatic, and advanced hepatocellular cancer [24]. Thus, MACC1 might be employed as a routine biomarker for diagnosis, disease prognosis and prediction of therapy response in the clinic. Tissue- and blood-based diagnostic tests have already been performed in retrospective and prospective studies [24].

However, the expression pattern of MACC1 protein within heterogeneous tumors with respect to refinement of patient risk assessment has not been addressed. Aim of this study is therefore to evaluate the geographic expression pattern of MACC1 protein in the tumor center, the invasion front and in tumor buds of clinical CRC samples. In parallel, we determined mismatch repair (MMR)-status, BRAF/KRAS-mutations and CpG-island methylation to determine the impact of oncogenic driver mutations on MACC1 expression. Taken together, we report for the first time the differential expression of MACC1 in CRC with increasing levels from tumor center to invasion front. MACC1 expression at the invasion front was identified as the best predictor for aggressive clinicopathological features, tumor budding, metastasis formation and poor survival outcome.

## Methods

### Patients and study design

**Figure 1:**
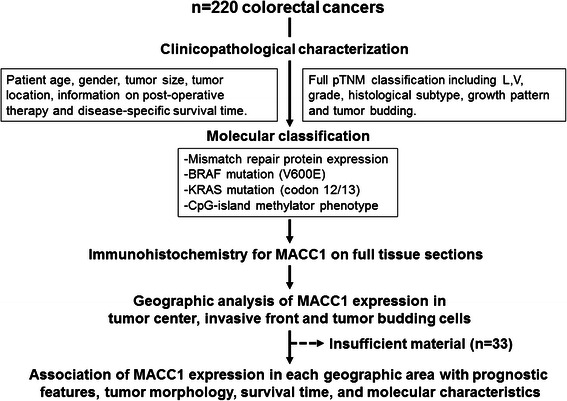
**Study design.** 220 CRC patients with full clinicopathological features were entered into the study. Cases were analyzed for BRAF and KRAS mutations and MMR-protein expression was determined. Tumors of the CpG-Island methylator phenotype were identified using pyrosequencing. MACC1 protein expression in normal mucosa, tumor center, tumor front and tumor buds was evaluated by immunohistochemistry using full tissue sections. MACC1 expression in each geographic area of CRC was analyzed with clinicopathological features, patient survival and molecular features.

Two hundred and twenty unselected, non-consecutive CRC patients surgically treated from 2004–2007 at the Aretaieion University Hospital, University of Athens, Greece were included in this study [Figure 1]. Clinical information on patient gender, age at diagnosis, tumor diameter, tumor location, post-operative therapy and disease-specific survival time was obtained from patient records. An experienced gastrointestinal pathologist (EK) reviewed all histopathological slides according to the UICC TNM Classification 7^th^ edition. Data on pathological T (pT), N (pN), and M-stage (pM), the presence of lymphatic invasion (L), venous invasion (V), perineural invasion (Pn), tumor grade (G), histological subtype and tumor growth pattern was recorded. Tumor budding was assessed using the 10 high-power fields (10HPF) method (40×; HPF field area 0.049 mm^2^) of highest density along the invasive front [25,26]. For each case, one full tissue section of invasive adenocarcinoma including the geographic areas tumor center, invasive front and tumor buds were selected for analysis of MACC1 expression by immunohistochemistry. Peritumoral normal mucosa was evaluated for MACC1 expression where available (n = 59). 33 cases were excluded based on insufficient material remaining on the tissue block. Final patient number was 187. Patient characteristics are found in [Table 1]. This study was designed in accordance with the reporting recommendations for tumor marker prognostic studies (REMARK) criteria [27].

**Table 1:** **Patient characteristics and association of MACC1 expression in the tumor center with clinicopathological data**

Characteristics	Total (n = 187)	MACC1 tumor center N (%); (n = 187)		P-value
		Low (Score 0)	High (Score 1–3)	
		N = 78 (41.7%)	N = 109 (58.3%)	
**Age (yrs.)**				
Mean (min, max)	68.6 (35–93)	69.2 (36–93)	68.1 (35–91)	0.1732
**Tumor size (cm)**				
Mean (min, max)	4.5 (1.2–12)	4.8 (2–12)	4.3 (1.2–8)	0.5723
**Gender**				
Male	88 (47.3)	35 (44.9)	53 (49.1)	0.5711
Female	98 (52.7)	43 (55.1)	55 (50.9)	
**Histological subtype**				
Non-mucinous	167 (89.3)	70 (89.7)	97 (89.0)	0.8695
Mucinous	20 (10.7)	8 (10.3)	12 (11.0)	
**Tumor grade**				
G1-2	120 (64.2)	55 (70.5)	65 (59.6)	0.126
G3	67 (35.8)	23 (29.5)	44 (40.4)	
**Tumor location**				
Left	113 (60.7)	42 (53.9)	71 (65.7)	0.2027
Rectum	21 (11.3)	9 (11.5)	12 (11.1)	
Right	52 (28.0)	27 (34.6)	25 (23.2)	
**pT**				
pT1 + pT2	47 (25.1)	26 (33.3)	21 (19.3)	0.0288
pT3 + pT4	140 (74.9)	52 (66.7)	88 (80.7)	
**pN**				
pN0	97 (51.9)	51 (65.4)	46 (42.2)	0.0018
pN1-2	90 (48.1)	27 (34.6)	63 (57.8)	
**pM**				
pM0	167 (89.8)	73 (93.6)	94 (87.0)	0.1454
pM1	19 (10.2)	5 (6.4)	14 (13.0)	
**TNM stage**				
Stage I	40 (21.5)	26 (33.3)	14 (13.0)	0.0023
Stage II	53 (28.5)	24 (30.8)	29 (26.9)	
Stage III	74 (39.8)	23 (29.5)	51 (47.2)	
Stage IV	19 (10.2)	5 (6.4)	14 (13.0)	
**Tumor budding**				
Low-grade	101 (54.0)	54 (69.2)	47 (43.1)	0.0004
High-grade	86 (46.0)	24 (30.8)	62 (56.9)	
**Venous invasion**				
Present	32 (17.1)	8 (10.3)	24 (22.0)	0.0352
Absent	155 (82.9)	70 (89.7)	85 (78.0)	
**Lymphatic invasion**				
Present	74 (39.6)	24 (30.8)	50 (45.9)	0.0373
Absent	113 (60.4)	54 (69.2)	59 (54.1)	
**Therapy**				
Untreated	66 (35.3)	39 (50.0)	27 (24.8)	0.0004
Treated	121 (64.7)	39 (50.0)	82 (75.2)	
**MMR status**				
Proficient	170 (91.4)	71 (91.0)	99 (91.7)	0.8777
Deficient	16 (8.6)	7 (9.0)	9 (8.3)	
**KRAS status**				
Wild-type	124 (67.0)	54 (70.1)	70 (64.8)	0.4484
Mutation	61 (33.0)	23 (29.9)	38 (35.2)	
**BRAF status**				
Wild-type	165 (91.2)	69 (92.0)	96 (90.6)	0.7378
Mutation	16 (8.8)	6 (8.0)	10 (9.4)	
**CIMP status**				
Negative/Low	90 (87.4)	40 (90.9)	50 (84.8)	0.3887
High	13 (12.6)	4 (9.1)	9 (15.3)	
**Survival rate**				
Median	60 (50-ne)	Not reached	58 (43-ne)	0.2585

ne = survival endpoint not reached.

### Ethics committee approval

The use of patient data has been approved by the Ethics Committee at the University of Athens, Greece.

### Tissue sections and MACC1 immunohistochemistry

**Figure 2:**
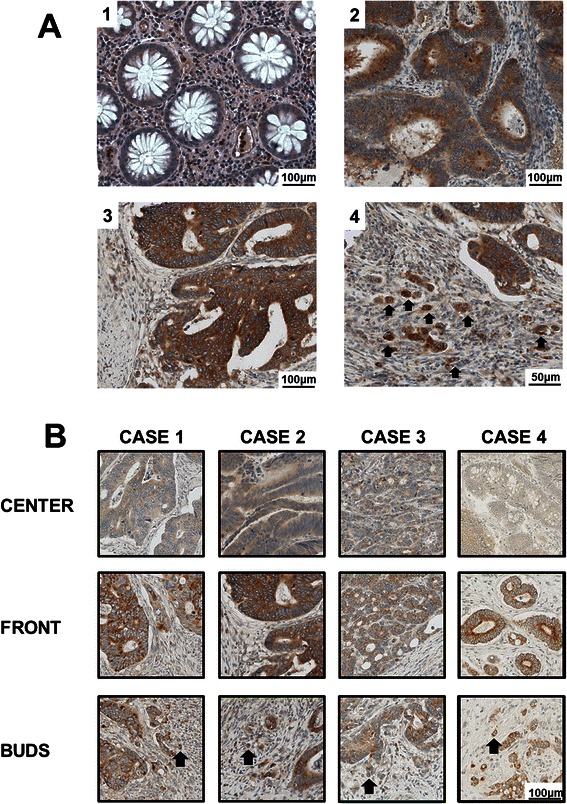
**MACC1 protein expression analysis in CRC. A**: MACC1 expression in normal mucosa (1), tumor center (2), tumor front (3) and tumor buds (4; arrows) was evaluated by immunohistochemistry. **B**: Four representative cases of colorectal adenocarcinoma showing a significant increase of MACC1 expression from the tumor center towards the invasive front and MACC1 over-expressing tumor budding cells.

Full tissue sections from formalin-fixed, paraffin-embedded surgical resection specimens were cut at 4 μm. For immunohistochemistry of MACC1, sections were deparaffinized by successive immersions in xylene (20 minutes), acetone/Tris 2:1, acetone/Tris 1:2, Tris/NaCl, aqua dest (5 minutes each). Epitopes were demasked with 10 mM citrate buffer (pH 6, microwave). After blocking (5% goat serum, 30 minutes), sections were incubated with the rabbit polyclonal anti-MACC1 antibody (1:100, Sigma HPA020103) for three hours at room temperature. Detection was performed using the biotin-based ABC kit (Dako; anti-rabbit biotin antibody and anti-biotin-streptavidin-HRP) and diaminobenzidine (1 minute) as substrate. Counter staining with Mayer’s haematoxylin was done for 2 minutes. Negative biological controls were performed using a matched multi-punch tissue microarray (TMA) of 50 CRC cases including normal mucosa [Figure 2A] and tumor tissue, negative technical controls were carried out by omitting the primary MACC1 antibody [Additional file 1: Figure S1].

### Evaluation of MACC1

We analyzed MACC1 expression in each geographic zone (normal mucosa, tumor center, invasive front) of whole tissue sections in analogy to the Rüschoff criteria for evaluation of Her2 biomarker expression [28]. Briefly, MACC1 expression was scored from 0 (absent staining) to 3 (strong staining). A score of 3 was assigned when a strong, unequivocally positive cytoplasmic and/or nuclear staining was observed at low magnification (5×) in a given geographic area. A score of 2 was assigned when higher magnification (10×) was needed to recognize MACC1 positivity. When high-power magnification (20×-40×) was required to recognize MACC1 positivity, a score of 1 was assigned. For tumor buds, the total number of buds was counted in one HPF of highest density at the invasive front and the number and proportion of buds showing MACC1 positivity was recorded.

### KRAS, BRAF and MMR status

BRAF (exon 15, V600E mutations) and KRAS (exon 2, codon 12 and 13) mutations were analyzed using pyrosequencing as previously described [29]. For identification of tumors with high-level CpG island methylation (CpG island methylator phenotype, CIMP), PCR analysis of CpG-loci of six genes (SOCS1, NEUROG1, MLH1, CRABP1A, CDKN2A, RUNX3) was carried out by pyrosequencing as recently reported [29].

Mismatch-repair (MMR) protein expression was determined by immunohistochemistry for MLH1, MSH2, MSH6, and PMS2 using a multi-punch tissue microarray containing an average of four tumor cores per case. Staining was carried out as previously described. MMR-protein expression was scored as positive when staining for all MMR-proteins was observed.

### Statistical analysis

MACC1 positive cases were defined as MACC1 scores 1–3 by immunohistochemistry, negative cases were defined as score 0. Differences in MACC1 expression by geographic area and tissue type were analyzed using the Kruskal-Wallis test. The correlation of MACC1 expression with clinicopathological and molecular features was evaluated using the Chi-Square, or Fisher’s Exact test as appropriate. Survival time analysis was performed using Kaplan-Meier curves and tested using the log-rank test in univariate analysis. Multivariate analysis for the prognostic effect of MACC1 expression at the tumor front and the potential confounders pT, pN, pM and adjuvant therapy was performed using a Cox regression model after verification of the proportional hazards assumption. Adjustment for multiple hypothesis testing was not undertaken [30]. P-values <0.05 were considered statistically significant. Analyses were performed using SAS (V9.2, The SAS Institute, Cary, NC).

## Results

### Geographic analysis of MACC1 expression

MACC1 was significantly over-expressed in tumor tissue as compared to normal mucosa (p < 0.001) [Figure 2A]. In tumor tissue, a gradient of MACC1 expression from the tumor center to the invasive front was identified (p = 0.0012) [Figure 2B]. In tumor buds, a strong cytoplasmic expression was observed: 55% of the dissociated single cells or small clusters of up to five cells identified at the invasive front demonstrated MACC1 expression [Figure 2B]. No MACC1 expression was observed in the tumor stroma.

### MACC1 expression in the tumor center

In the tumor center, MACC1 expression (score 1–3) was observed in 58.3% of cases. Patients with strong MACC1 expression in the tumor center frequently presented with locally advanced pT3/4 tumors (p = 0.0288) as compared to MACC1 negative cases [Table 1]. In fact, 80% of MACC1 positive tumors showed infiltration into the pericolic fat or penetration of the serosa. MACC1 positivity in the tumor center further predicted aggressive tumor growth with presence of lymphatic invasion (p = 0.0373), venous invasion (p = 0.0352) and frequent metastasis to loco regional lymph nodes (p = 0.0018). Further, MACC1 expression in the tumor center was highly correlated with presence of high-grade tumor budding. However, no impact of MACC1 expression in the tumor center on the frequency of distant metastasis or patient survival was observed.

### MACC1 expression at the invasive front

**Figure 3:**
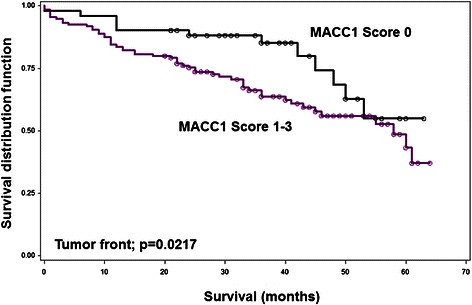
**Prognostic effects of MACC1 expression at the invasive front of CRC.** MACC1 overexpression at the tumor front is a significant adverse prognostic indicator (p = 0.0217) in univariate survival analysis.

At the tumor front, MACC1 expression was observed in 72.2% of cases. MACC1 staining at the tumor front was seen in aggressive tumors with more advanced pT-stage (p = 0.0005), presence of lymphatic (p = 0.002) and venous (p = 0.0125) invasion as well as frequent nodal metastasis (p = 0.0001) [Table 2]. MACC1 expression at the tumor front was strongly predictive for the formation of distant metastasis (p = 0.0223). In fact, 18 of 19 patients with distant metastasis were correctly identified based on marker expression in this geographic area, indicating that MACC1 expression at the tumor host interface may be particularly important for tumor spread to distant organs. In consistence with this, strong MACC1 expression at the invasive front correlated with a high grade tumor budding phenotype (p = 0.0006). MACC1 positivity at the invasive front predicted poor overall survival outcome (p = 0.0217) in univariate analysis [Figure 3], however the prognostic impact of marker expression was not independent of T-stage, N-stage and adjuvant therapy as identified in multivariable analysis (p = 0.7827) [Table 3].

**Table 2:** **Association of MACC1 expression in the tumor front and tumor buds with clinicopathological data**

Characteristics	MACC1 tumor front N (%); (n = 187)		P-value	MACC1 tumor buds N (%); (n = 187)		P-value
	Low (Score 0)	High (Score 1–3)		Low (<median)	High (>median)	
**Age (yrs.)**						
Mean (min, max)	69.9 (38–88)	68.1 (35–93)	0.1141	70.1 (36–89)	67.6 (41–91)	0.0408
**Tumor size (cm)**						
Mean (min, max)	5.0 (2–12)	4.4 (1.2–8.0)	0.4457	4.6 (2–12)	4.5 (1.2–8)	0.7134
**Gender**						
Male	18 (35.3)	70 (51.9)	0.0436	23 (46.0)	32 (45.1)	0.9195
Female	33 (64.7)	65 (48.2)		27 (54.0)	39 (54.9)	
**Histological subtype**						
Non-mucinous	46 (90.2)	121 (89.0)	0.8092	45 (90.0)	61 (85.9)	0.502
Mucinous	5 (9.8)	15 (11.0)		5 (10.0)	10 (14.1)	
**Tumor grade**						
G1-2	36 (70.6)	84 (61.8)	0.2624	30 (60.0)	35 (49.3)	0.2449
G3	15 (29.4)	52 (38.2)		20 (40.0)	36 (50.7)	
**Tumor location**						
Left	27 (52.9)	86 (63.7)	0.3547	30 (60.0)	43 (61.4)	0.7867
Rectum	6 (11.8)	15 (11.1)		7 (14.0)	7 (10.0)	
Right	18 (35.3)	34 (25.2)		13 (26.0)	20 (28.6)	
**pT**						
pT1 + pT2	22 (43.1)	25 (18.4)	0.0005	15 (30.0)	3 (4.2)	<0.0001
pT3 + pT4	29 (56.9)	111 (81.6)		35 (70.0)	68 (95.8)	
**pN**						
pN0	38 (74.5)	59 (43.4)	0.0001	26 (52.0)	24 (33.8)	0.0453
pN1-2	13 (25.5)	77 (56.6)		24 (48.0)	47 (66.2)	
**pM**						
pM0	50 (98.0)	117 (86.7)	0.0223	47 (94.0)	60 (85.7)	0.1499
pM1	1 (2.0)	18 (13.3)		3 (6.0)	10 (14.3)	
**TNM stage**						
Stage I	22 (43.1)	18 (13.3)	<0.0001	13 (26.0)	1 (1.4)	0.0004
Stage II	15 (29.4)	38 (282.)		12 (24.0)	20 (28.6)	
Stage III	13 (25.5)	61 (45.2)		22 (44.0)	39 (55.7)	
Stage IV	1 (2.0)	18 (13.3)		3 (6.0)	10 (14.3)	
**Tumor budding**						
Low-grade	38 (74.5)	63 (46.3)	0.0006	28 (56.0)	20 (28.2)	0.0021
High-grade	13 (25.5)	73 (53.7)		22 (44.0)	51 (71.8)	
**Venous invasion**						
Present	3 (5.9)	29 (21.3)	0.0125	7 (14.0)	19 (26.8)	0.0924
Absent	48 (94.1)	107 (78.7)		43 (86.0)	52 (73.2)	
**Lymphatic invasion**						
Present	11 (21.6)	63 (46.3)	0.002	24 (48.0)	38 (53.5)	0.5496
Absent	40 (78.4)	73 (53.7)		26 (52.0)	33 (46.5)	
**Therapy**						
Untreated	29 (56.9)	37 (27.2)	0.0002	24 (48.0)	9 (12.7)	<0.0001
Treated	22 (43.1)	99 (72.8)		26 (52.0)	62 (87.3)	
**MMR status**						
Proficient	44 (86.3)	126 (93.3)	0.1256	46 (92.0)	63 (90.0)	0.7607
Deficient	7 (13.7)	9 (6.7)		4 (8.0)	7 (10.0)	
**KRAS status**						
Wild-type	32 (64.0)	92 (68.2)	0.594	33 (66.0)	46 (65.7)	0.974
Mutation	18 (36.0)	43 (31.9)		17 (34.0)	24 (34.3)	
**BRAF status**						
Wild-type	45 (90.0)	120 (91.6)	0.772	44 (89.8)	60 (89.6)	0.966
Mutation	5 (10.0)	11 (8.4)		5 (10.2)	7 (10.5)	
**CIMP status**						
Negative/Low	27 (90.0)	63 (86.3)	0.7514	21 (84.0)	36 (83.7)	1.0
High	3 (10.0)	10 (13.7)		4 (16.0)	7 (16.3)	
**Survival rate**						
Median	Not reached	58 (43-ne)	0.0217	53.0 (43–61)	55.0 (36-ne)	0.839

ne = survival endpoint not reached.

**Table 3:** **Multivariable Cox**-**regression analysis of MACC1 expression at the tumor front TNM**-**stage and adjuvant therapy**

Parameter		HR (95%CI)	P-value
**MACC1**			
Low (Score 0)	1.0	0.7827	
High (Score 1–3)	1.1 (0.56-2.16)		
**pT**			
pT1-2	1.0	0.3425	
pT3-4	1.52 (0.64-3.57)		
**pN**			
pN0	1.0	0.0003	
pN1-2	3.48 (1.77-6.85)		
**pM**			
pM0	1.0	<0.0001	
pM1	4.12 (2.2-7.68)		
**Adjuvant therapy**			
None	1.0	0.3284	
Treated	0.74 (0.4-1.36)		

### MACC1 expression in tumor buds

MACC1 expression was observed in 55% of tumor buds. In tumor buds, MACC1 expression correlated with aggressive disease biology. A higher proportion of MACC1 positive tumor buds was detected in patients with more advanced T-stage (p < 0.0001), higher overall TNM-stage (p = 0.0004) and presence of nodal metastasis (p = 0.0453) [Table 2]. No impact of MACC1 expression in tumor buds on survival was observed.

### Geographic expression patterns of MACC1 in a molecular pathology context

KRAS mutations were identified in 32.6% of patients (n = 61) by pyrosequencing, while activating BRAF (V600) mutations were found in 8.5% of patients (n = 16). 8.5% of patients showed loss of MMR-protein expression by immunohistochemistry, while 7% (n = 13) of cases were classified as CIMP-high. No impact of molecular features on MACC1 protein expression was observed independent of the geographic area analyzed.

## Discussion

In the current study we perform a geographic analysis of MACC1 expression in CRC with a particular focus on EMT-like cancer cells in the tumor microenvironment, also called tumor buds. As molecular features of CRC impact prognosis, we correlate MACC1 protein expression with MMR-status, BRAF- and KRAS-mutation as well as CpG-island methylation.

We demonstrate that MACC1 is variably expressed in normal mucosa, tumor center, invasive front and tumor buds. CRC is a highly heterogeneous disease characterized by marked genetic, spatial and temporal dynamics [31]. Tumor heterogeneity is present even in early invasive disease and may affect the reproducibility of biomarker assessment [32]. In a novel geographic approach towards immunohistochemical MACC1 expression analysis on full tissue sections, we identify a progressive increase of MACC1 positivity from the tumor center towards the invasive front with frequent overexpression in tumor budding cells. MACC1 gene function has been implicated in disease progression of CRC through activation of the MET- and beta-catenin (CTNNB1) pathways [3,33]. Activation of EMT in the process of invasion is a central step towards the seeding of metastasis [34-36]. Importantly, MACC1 overexpression at the invasive front was significantly associated with presence of distant metastasis and was a strong prognostic indicator. As death from CRC is predominantly determined by metastatic dissemination, the prognostic impact of MACC1 in this geographic area further corroborates the exceptional importance of the tumor microenvironment for determining prognosis [37].

Tumor budding is officially recognized by the Union for International Cancer Control (UICC) as an independent additional prognostic indicator in CRC [38]. A high grade tumor budding phenotype is consistently associated with aggressive clinicopathological features, lymph node and distant metastasis [36]. It is thought that tumor budding at the tumor invasive front is a histomorphological hallmark of EMT. Tumor buds overexpress protein markers associated with tumor cell migration and invasion such as matrix metallopeptidase 2 (MMP2), MMP9 and cathepsinB (CTSB) [36]. Interestingly, MMP9 was previously described as being regulated by MACC1 in hepatocellular carcinoma and gastric cancer cell lines [39,40]. Further, activation of WNT-signaling and loss of E-Cadherin (CDH1) contributes to dissociative growth of tumor budding cells and loss of an epithelial phenotype. The expression of proteins such as Raf-kinase inhibitor protein (RKIP) and neurotrophic tropomyosine kinase receptor type 2 (NTRK2) contribute to the resistance to apoptosis and anoikis [41,42]. Interestingly, MACC1 overexpression in any geographic region of CRC was significantly associated with high grade tumor budding at the invasive front and aggressive histopathological features including more advanced pT and pN-stages, venous and lymphatic invasion. In consistence with recently published literature, this suggests that active MET signaling contributes to dissociative tumor growth, tumor progression and invasion [43]. MACC1 overexpression in tumor budding cells themselves provides further evidence of their biological aggressiveness and likens these cells to EMT-like cancer cells [43]. As MACC1 has been suggested as a potential therapeutic target, overexpression on EMT-like cancer cells may represent an attractive option to manipulate cancer initiating cells at the tumor host interface in the process of invasion [44].

Biomarkers with predictive value for metastatic disease relapse have the potential to aid clinical management of CRC patients as additional prognostic indicators. The current approach towards active surveillance of CRC patients following resection is monitoring of serum carcinoembryonic antigen, but suffers from suboptimal sensitivity and specificity [45]. As metastatic relapse is a decisive event that determines prognosis of the CRC patient, early identification of high risk patients is an important goal for biomarker development. Several biomarkers have recently been highlighted to guide the identification of patients at risk of metastatic relapse. Examples include expression of RKIP in the primary tumor [41] and serum biomarkers such as microRNA-200c [46]. Based on the variety of detection methods, possibilities for assessment in tumor tissue and plasma and inclusion in early clinical trials, MACC1 is a promising candidate in the growing list of potentially valuable biomarkers to aid the identification of high risk CRC patients [6,24].

Molecular markers such as KRAS- and BRAF-mutations contribute predictive information for response to EGFR-inhibitors, but their value for identification of patients at high risk of metastatic relapse independent of disease stage is limited [47]. Interestingly, MACC1 expression was found to be independent of oncogenic driver mutations including activating KRAS-, BRAF- mutations, microsatellite instability and CIMP. This suggests that the association of MACC1 overexpression with presence of metastatic disease may be independent of the genetic features of CRC. MACC1 may therefore represent a complementary biomarker to KRAS- and BRAF- gene mutation status allowing the identification of patients at high risk of metastatic relapse. However, based on the relatively small number of cases identified with KRAS-, BRAF- mutations, CIMP or MMR-deficiency, this data cannot exclude an association between MACC1 and the molecular markers under study and requires independent validation.

This investigation has several strengths. The study is designed based on a hypothesis driven approach in full accordance with the REMARK guidelines for tumor marker prognostic studies [27]. Analyses are based on a very well characterized cohort of 187 CRC patients with full clinicopathological data, follow-up and therapy information. Marker analysis on full tissue sections accounts for tumor heterogeneity and allows expression analysis in tumor buds at the tumor-host interface. Weaknesses include the relatively small patient number included in the analysis of molecular pathology features with MACC1 expression. Further, marker cut-off levels may be influenced by the analysis methods and specific characteristics of the cohort under study. Consequently we recommend validation of the geographic expression pattern of MACC1 as a biomarker using independent patient cohorts.

## Conclusions

This study further advances the development of MACC1 as a predictive biomarker. By geographic protein expression analysis, we illustrate that MACC1 is differentially expressed in normal mucosa, tumor center and at the invasive front of colorectal cancer. Marker positivity is frequently seen in tumor buds and identifies cancer cells with particularly aggressive behavior. At the invasive front, MACC1 expression best predicts aggressive clinicopathological features, tumor budding, and metastasis formation. MACC1 biomarker expression was not influenced by MMR-status, BRAF or KRAS-mutations or CpG-island methylation. Based on meaningful functional data and strong potential for translational application, MACC1 has to be classified as a promising biomarker for validation in prospective studies.

## Additional file

Additional file 1: Figure S1. Technical controls. No immune reactivity was observed in technical controls of normal mucosa (A), tumor center (B), tumor front (C) and tumor buds (D; arrows).

## Abbreviations

EMT: Epithelial-mesenchymal transition
CDH1: E-Cadherin
CRC: Colorectal cancer
CTNNB1: Beta-catenin
CTSB: CathepsinB
CIMP: CpG-island methylator phenotype
HPF: High-power field
MACC1: Metastasis associated in colon cancer 1
MET: MET proto-oncogene, receptor tyrosine kinase
MMP: Matrix metallopeptidase
MMR: Mismatch repair
NTRK2: Neurotrophic tropomyosine kinase receptor type 2
REMARK: Reporting recommendations for tumor marker prognostic studies
RKIP: Raf kinase inhibitor protein
TMA: Tissue microarray
UICC: Union for International Cancer Control
