# Supplementary figures and images for: Heterogeneity analysis of Metastasis Associated in Colon Cancer 1 (MACC1) for survival prognosis of colorectal cancer patients: a retrospective cohort study
Source: BMC Cancer. 2015 Mar 21;15:160. doi: 10.1186/s12885-015-1150-z (PMC4371627; doi:10.1186/s12885-015-1150-z)

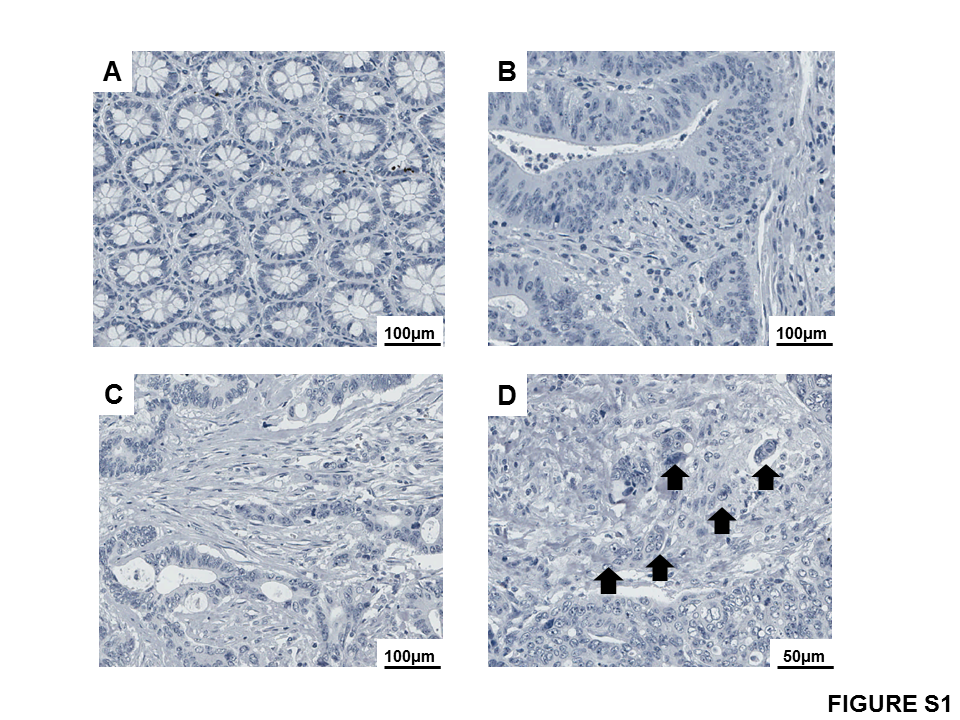

Supplement: Additional file 1: Figure S1. — Technical controls. No immune reactivity was observed in technical controls of normal mucosa (A), tumor center (B), tumor front (C) and tumor buds (D; arrows). [file 12885_2015_1150_MOESM1_ESM.tiff]
